# Sharpin promotes hepatocellular carcinoma progression via transactivation of Versican expression

**DOI:** 10.1038/oncsis.2016.76

**Published:** 2016-12-12

**Authors:** Y Tanaka, K Tateishi, T Nakatsuka, Y Kudo, R Takahashi, K Miyabayashi, K Yamamoto, Y Asaoka, H Ijichi, R Tateishi, J Shibahara, M Fukayama, T Ishizawa, K Hasegawa, N Kokudo, K Koike

**Affiliations:** 1 Department of Gastroenterology, Graduate School of Medicine, The University of Tokyo, Tokyo, Japan; 2 Department of Pathology, Graduate School of Medicine, The University of Tokyo, Tokyo, Japan; 3 Hepato-Biliary-Pancreatic Surgery Division, Department of Surgery, Graduate School of Medicine, The University of Tokyo, Tokyo, Japan

## Abstract

Sharpin (Shank-associated RH domain-interacting protein, also known as SIPL1) is a multifunctional molecule that participates in various biological settings, including nuclear factor-κB signaling activation and tumor suppressor gene inhibition. Sharpin is upregulated in various types of cancers, including hepatocellular carcinoma (HCC), and is implicated in tumor progression. However, the exact roles of Sharpin in tumorigenesis and tumor progression remain largely unknown. Here we report novel mechanisms of HCC progression through Sharpin overexpression. In our study, Sharpin was upregulated in human HCC tissues. Increased Sharpin expression enhanced hepatoma cell invasion, whereas decrease in Sharpin expression by RNA interference inhibited invasion. Microarray analysis identified that Versican, a chondroitin sulfate proteoglycan that plays crucial roles in tumor progression and invasion, was also upregulated in Sharpin-expressing stable cells. Versican expression increased in the majority of HCC tissues and knocking down of Versican greatly attenuated hepatoma cell invasion. Sharpin expression resulted in a significant induction of Versican transcription synergistically with Wnt/β-catenin pathway activation. Furthermore, Sharpin-overexpressing cells had high tumorigenic properties *in vivo*. These results demonstrate that Sharpin promotes Versican expression synergistically with the Wnt/β-catenin pathway, potentially contributing to HCC development. A Sharpin/Versican axis could be an attractive therapeutic target for this currently untreatable cancer.

## Introduction

Hepatocellular carcinoma (HCC) is one of the most frequently diagnosed cancers worldwide. Long-term survival rates of patients with HCC remain poor even after curative resection or radiofrequency ablation therapy.^1^ A major cause of high mortality is the development of vascular invasion features such as portal vein tumor thrombus.^2^ The 5-year survival rate after vascular invasion development was only 6.4% in our institute.^3^ The molecular mechanisms underlying HCC invasion remain ill-defined, hindering the development of novel therapeutic options. Better elucidation of the molecular pathway involved in HCC invasion could facilitate the development of better treatment options for patients.

Sharpin (Shank-associated RH domain interacting protein), also known as SIPL1 (Shank-interacting protein-like 1), was first identified as a protein that directly interacts with the ankyrin repeats of Shank family proteins in the postsynaptic density.^4^ Another important role of Sharpin was later recognized as one of the components of an E3 ubiquitin–protein ligase complex, the linear ubiquitin chain assembly complex (LUBAC). LUBAC consists of HOIL-1 (heme-oxidized IRP2 ubiquitin ligase 1), HOIP (HOIL-1-interacting protein) and Sharpin. LUBAC also adds a linear polyubiquitin chain to NEMO/IKKγ, a regulatory subunit in the IkB kinase (IKK) complex, that consequently activates nuclear factor-κB (NF-κB) signaling.^5, 6, 7^ In addition, Sharpin is reported to bind to tumor suppressor PTEN (phosphatase and tensin homolog) and inhibit its lipid phosphatase activity to promote tumorigenesis.^8, 9^ Sharpin also acts as an endogenous inhibitor of integrin signaling to retard cell migration.^10^ Furthermore, Sharpin was shown to act as a coactivator for the homeobox protein, SIX homeobox 1 (SIX1), together with eye absent 1 protein (EYA1).^11^ These reports indicate that Sharpin is a multifunctional molecule that participates in various biological settings.

Recent studies have shown that Sharpin is frequently upregulated in multiple human cancer types, including ovarian, prostate and breast cancers.^12, 13, 14, 15^ Sharpin promotes cell survival, growth, and invasion, suggesting tumor-associated roles during cancer biogenesis. These functions are dependent on NF-κB signaling functions such as the induction of matrix metalloproteinases (MMP-2 and MMP-9) and upregulation of survivin, a member of the inhibitor of apoptosis family.^13, 16^ Sharpin expression also increases in HCC;^12^ however, the biological significance underlying HCC progression remains largely unknown.

In this study, we show that Sharpin expression is frequently upregulated in human HCC. Sharpin overexpression led to increased expression of Versican, an aggregating chondroitin sulfate proteoglycan that plays biological roles in tumor progression and invasion,^17, 18^ leading to HCC cell invasion. Our findings indicate the importance of a novel Sharpin/Versican axis in HCC invasion.

## Results

### Sharpin is frequently overexpressed in HCC

**Figure 1:**
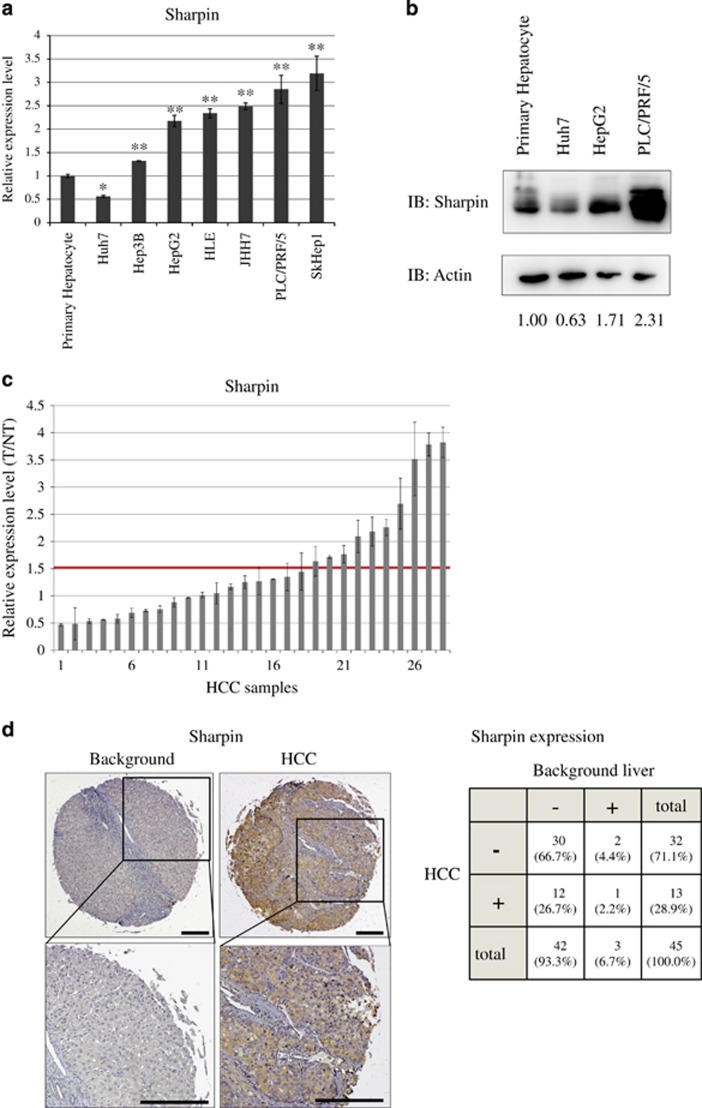
Sharpin expression is high in hepatocellular carcinoma. (**a**) Sharpin mRNA expression in hepatoma cell lines. Values were internally normalized by GAPDH mRNA. Data represent the mean±s.d. (*P<0.05, **P<0.01, two-tailed Student's *t*-test). (**b**) Sharpin protein expression in hepatoma cell lines. Numbers below the panels indicate Sharpin protein levels normalized by actin levels. (**c**) Sharpin mRNA expression in surgically resected HCC samples (T/NT, tumor/non-tumor ratio). Values were internally normalized by GAPDH mRNA (*n*=28). (**d**) Immunohistochemical analysis of Sharpin protein expression in HCC and surrounding tissues (background liver). The lower panels display magnified images of the boxed areas in the upper panels. The staining intensity was classified into two categories: no or faint staining was ranked as (−) (background in this case), and moderate or dark staining was ranked as (+) (HCC in this case). Representative case is shown. Scale bars, 100 μm. Grid summarizing Sharpin immunohistochemical staining data from 45 cases (right). Sharpin expression was significantly higher in HCC than in surrounding tissues (*P*<0.05, Wilcoxon signed-rank test).

The expression level of Sharpin mRNA in HCC cells was initially determined by quantitative RT–PCR. Sharpin expression was greater in most of the HCC cell lines than in the primary hepatocyte cells (Figure 1a). Increases in Sharpin protein expression were confirmed by western blotting in the HCC cell lines, HepG2, and PLC/PRF/5 (Figure 1b). Among all cell lines, only Huh7 cells had low Sharpin expression at both the mRNA and protein levels.

We then examined Sharpin expression in surgically resected HCC samples. Sharpin mRNA expression was 1.5-fold higher in 36% (10/28) of the surgically resected HCC clinical samples relative to that in noncancerous tissues (Figure 1c). Sharpin expression was positively correlated with larger HCC size and histological grading (*P*<0.05, Spearman's analysis of correlation; Table 1). Immunohistochemical analysis confirmed that Sharpin expression was mainly observed in the cytoplasm and was significantly higher in HCC than in surrounding noncancerous liver tissue (*P*<0.05, Wilcoxon signed-rank test; Figure 1d).

These results indicate that Sharpin expression frequently increases in human HCC and this increased Sharpin expression might be involved in the pathogenesis of a subset of HCC cases.

### Sharpin promotes HCC invasion

We established Sharpin-expressing stable Huh7 cells (Huh7-Sharpin), in which endogenous Sharpin was low (Figure 1a), and control Huh7 cells (Huh7-ctrl), using a lentivirus infection to investigate the biological consequences of Sharpin upregulation in HCC.

**Figure 2:**
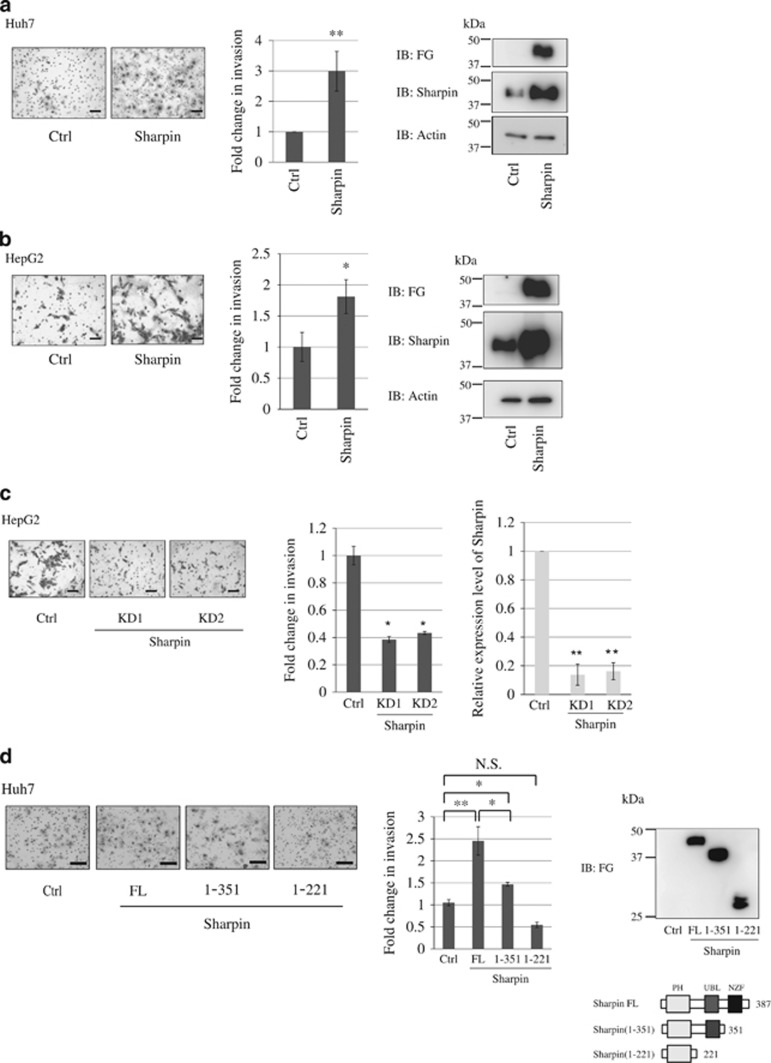
Sharpin promotes HCC invasion. (**a**) Increased expression of Sharpin-enhanced HCC cell invasion. Sharpin-expressing stable Huh7 cells (Huh7-Sharpin) and control cells (Huh7-ctrl) were established using a lentiviral vector and the degree of cell invasion was examined using Matrigel cell invasion chambers. Representative images of stained invaded cells (left). The relative cell invasion ratio after normalization to control cell invasion levels (middle). Data represent the mean±s.d. of three independent experiments. Scale bar, 100 μm (**P<0.01, two-tailed Student's *t*-test). Immunoblotting shows expression of FG-Sharpin protein in Sharpin-Huh7 cells (right). The molecular masses of the protein standards are indicated. (**b**) Sharpin-expressing stable HepG2 cells (HepG2-Sharpin) and control cells (HepG2-ctrl) were established using a lentiviral vector, and the degree of cell invasion was examined using Matrigel cell invasion chambers (middle) (*P<0.05, two-tailed Student's *t*-test). Representative images of stained invaded cells (left). Immunoblotting shows expression of FG-Sharpin protein in Sharpin-HepG2 cells (right). (**c**) Decreased Sharpin expression by RNA interference inhibited invasion of HCC cells. Endogenous Sharpin was knocked down in HepG2 cells and an invasion assay was performed 48 h later. Representative images of stained invaded cells (left). The relative cell invasion ratio after normalization to control cell invasion levels (middle). Expression level of Sharpin mRNA in HepG2 cells are shown (right). (**d**) UBL and NZF domains of Sharpin are indispensable for invasion of HCC cells. Full-length (FL) Sharpin and its deletion mutants Sharpin (1–351) and Sharpin (1–221) were established using lentiviral vectors and examined for HCC invasion (Huh7-Sharpin(1–351) and Huh7-Sharpin(1–221)) (middle). Representative images of stained invaded cells (left). Cell lysates were immunoblotted with the corresponding antibodies (right). The molecular masses of the protein standards are indicated. Scheme of Sharpin deletion mutants is shown (bottom) (NS, not significant; two-tailed Student's *t*-test).

We first performed a cell proliferation assay. However, no significant difference in cell growth was observed between Huh7-Sharpin and Huh7-ctrl cells (Supplementary Figure S1). We then performed invasion assays to characterize the invasive phenotype of Sharpin-expressing cells. Cell invasion rates were significantly increased in Huh7-Sharpin cells compared to that in Huh7-control cells (Figure 2a). By contrast, knockdown of Sharpin in Huh7 cells did not show significantly less invasive phenotype, possibly owing to low expression level of endogenous Sharpin in Huh7 cells (Supplementary Figure S2).

We also established Sharpin-expressing stable HepG2 cells, in which endogenous Sharpin level was high (Figure 1a). Sharpin-expressing stable HepG2 cells (HepG2-Sharpin) showed high invasive rates compared to HepG2-ctrl cells (Figure 2b). Notably, knockdown of Sharpin in HepG2 cells resulted in significantly lower rates of cell invasion (Figure 2c).

These results indicate that overexpression of Sharpin in HCC cells leads to increases in malignancy-related cellular properties, including invasion.

Deletion mutants of Sharpin were examined to further characterize the domain of Sharpin that is responsible for HCC invasion. Sharpin (1–351) lacks the NZF (Npl4 zinc finger) domain and Sharpin (1–221) lacks both the NZF and UBL (ubiquitin-like) domains, which are both important for NF-κB activation.^5^ The NZF domain deletion decreased HCC invasion to the control cell level and deletions of both the NZF and UBL domains of Sharpin markedly decreased HCC invasion (Figure 2d), showing that these domains of Sharpin are critical for HCC invasion.

Huh7-Sharpin and control cells were stimulated by TNFα to assess whether HCC invasion is the consequence of NF-κB activation; however, no significant invasion was observed (Supplementary Figure S3).

### Versican is upregulated in Sharpin-expressing stable cells

We performed complementary DNA microarray analyses to compare the expression profiles of Huh7-Sharpin cells to identify molecules responsible for invasion following Sharpin expression. Relative to the findings in control cells, 465 genes were downregulated (fold change <0.5) and 479 genes were upregulated (fold change >2.0) in Huh7-Sharpin cells.

**Figure 3:**
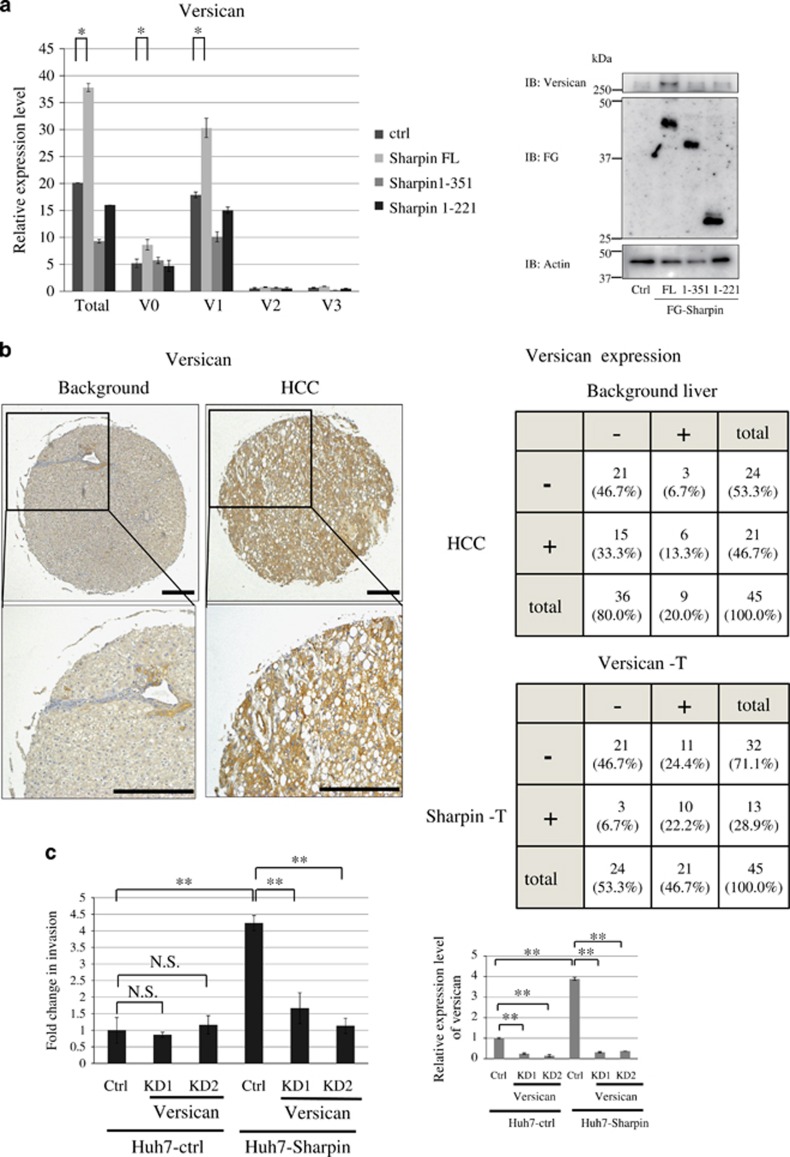
Versican is upregulated in Sharpin-expressing stable cells. (**a**) Versican expression in Huh7-Sharpin, Huh7-Sharpin(1–351) and Huh7-Sharpin(1–221), as examined by quantitative real-time PCR (left) and western blotting (right). Values were internally normalized by GAPDH mRNA (mean±s.d.; *P<0.05, two-tailed Student's *t*-test). (**b**) Immunohistochemical analysis of Versican protein expression in HCC and surrounding tissues (background liver). The lower panels display magnified images of the boxed areas in the upper panels. The staining intensity was classified into two categories: no or faint staining was ranked as (−) (background in this case), and moderate or dark staining was ranked as (+) (HCC in this case). Representative case is shown. Scale bars, 100 μm. Grid summarizing Versican immunohistochemical staining data from 45 cases. Versican was significantly overexpressed in HCC relative to surrounding tissues (*P*<0.05, Wilcoxon signed-rank test). Grid summarizing Sharpin and Versican immunohistochemical staining data in HCC from 45 cases. A positive correlation between Sharpin and Versican expression was observed in HCC (*P*<0.05, Fisher's exact probability test). (**c**) Increased expression of Versican enhanced invasion of HCC cells. Versican was knocked down in Huh7-Sharpin or Huh7-ctrl cells and an invasion assay was performed (left). Data represent the mean±s.d. of three independent experiments. Versican expression was determined by qRT-PCR (right) (NS, not significant; **P<0.01, two-tailed Student's *t*-test).

Contrary to our expectations, genes driven by NF-κB were not significantly upregulated in Sharpin-expressing cells. We searched for candidate genes to determine the mechanisms underlying enhanced invasion in Sharpin-expressing cells. We found that Versican expression was significantly increased when Sharpin was overexpressed (Table 2). The increased expression of total Versican in Huh7-Sharpin cells was confirmed via qRT-PCR and western blotting with pan-Versican antibody (Figure 3a). Levels of the V1 isoform were significantly higher in tumor tissues than in normal tissue, as previously reported.^19^ The other Versican isoforms, V2 and V3, were expressed at significantly lower levels in the liver (Figure 3a).

Immunohistochemical analysis confirmed total Versican expression in the extracellular matrix and cytoplasm, consistent with the results of a previous report^20^ (Figure 3b). Versican expression was significantly higher in HCC than in surrounding noncancerous liver tissue (*P*<0.05, Wilcoxon signed-rank test). Furthermore, a positive correlation between Sharpin and Versican expression was observed in HCC (*P*<0.05, Fisher's exact probability test; Figure 3b).

We knocked down Versican in Huh7-Sharpin cells to assess if it is involved in Sharpin-mediated HCC invasion. Cell invasion rates were significantly lower in Versican knockdown cells than in Huh7-Sharpin cells (Figure 3c). Knocking down of Versican in HepG2 cells also attenuated HCC invasion (Supplementary Figure S4), indicating that Versican plays a role in Sharpin-mediated HCC invasion.

### Sharpin transactivates Versican expression

Sharpin is reported to act as a coactivator of a transcription factor;^11^ therefore, we examined the effect of Sharpin on Versican expression.

**Figure 4:**
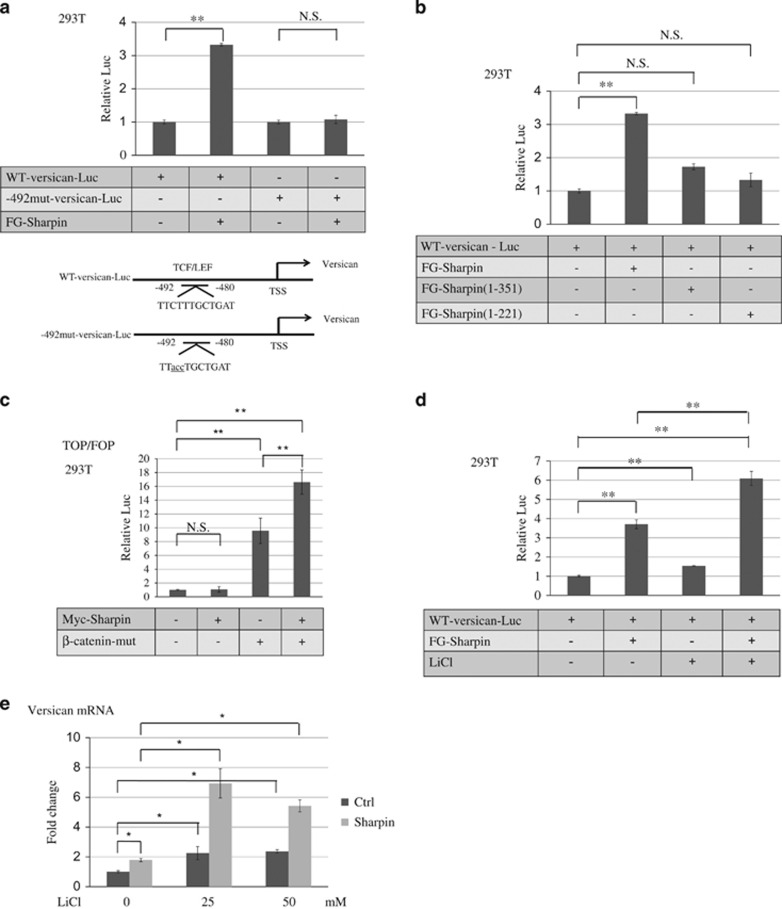
Sharpin transactivates Versican expression. (**a**) Sharpin activates Versican transcription through the TCF/LEF binding region. 293 T cells were cotransfected with WT-Versican-luc/-492 mut-Versican-luc and FG-Sharpin expression vector. Cell lysates were collected 48 h later and luciferase assays were performed. Values were internally normalized to *Renilla* (mean±s.d.; **P<0.01, NS, not significant, two-tailed Student's *t*-test). (**b**) NZF and UBL domains of Sharpin are indispensable for Versican transcription. HEK293T cells were transiently transfected with full-length Sharpin and its deletion mutants of Sharpin together with WT-Versican-luc. Cell lysates were collected 48 h later and luciferase assays were performed. (**c**) Sharpin synergistically activates TOP/FOP system with the Wnt pathway. HEK293T cells were transiently transfected with Sharpin and/or β-catenin together with TOP/FOP flash plasmids. Cell lysates were collected 48 h later and luciferase assays were performed. (**d**) Sharpin synergistically activates Versican transcription with the Wnt pathway. HEK293T cells were transiently transfected with FL Sharpin together with WT-Versican-luc and simultaneously stimulated with LiCl for 48 h. Cell lysates were collected and luciferase assays were performed. (**e**) Sharpin synergistically activates Versican mRNA with Wnt pathway activation. Huh7-Sharpin or Huh7-ctrl cells were stimulated with LiCl for 48 h and Sharpin mRNA expression was examined by qRT-PCR (*P<0.05, two-tailed Student's *t*-test).

To investigate the effect of Sharpin on Versican transcription, 293 T cells were cotransfected with a Versican promoter and Sharpin expression plasmids and then assayed for luciferase activity. The introduction of Sharpin induced significant Versican promoter activity when the wild-type Versican promoter was used. Sharpin did not induce Versican promoter activity when the −492 mutant Versican promoter that was mutated in a putative TCF/LEF-binding site localized at a position −492 bp from the transcription start site was used (Figure 4a).^21^ We found that the NZF domains of Sharpin were responsible for Versican expression (Figure 4b). As previously reported, introduction of Sharpin–HOIP, but not Sharpin alone, induced NF-κB transcriptional activity (Supplementary Figure S5),^5^ suggesting that this Versican upregulation may occur through NF-κB-independent mechanisms.

We employed the TOP/FOP reporter system using the dual-luciferase kit to assess the transcriptional activity of β-catenin and Sharpin. The introduction of Sharpin alone did not induce significant TOP/FOP reporter activation. However, the cotransfection of Sharpin and β-catenin synergistically increased reporter activity more than β-catenin alone (Figure 4c). LiCl, a GSK-3β inhibitor that leads to increased expression of β-catenin protein and the activation of the Wnt/β-catenin pathway, synergistically increased both Sharpin-induced Versican promoter activation (Figure 4d) and Versican mRNA expression (Figure 4e).

These data indicate that Sharpin-induced Versican transcription acts synergistically with the Wnt/β-catenin pathway.

### Sharpin may act as a coactivator for β-catenin

**Figure 5:**
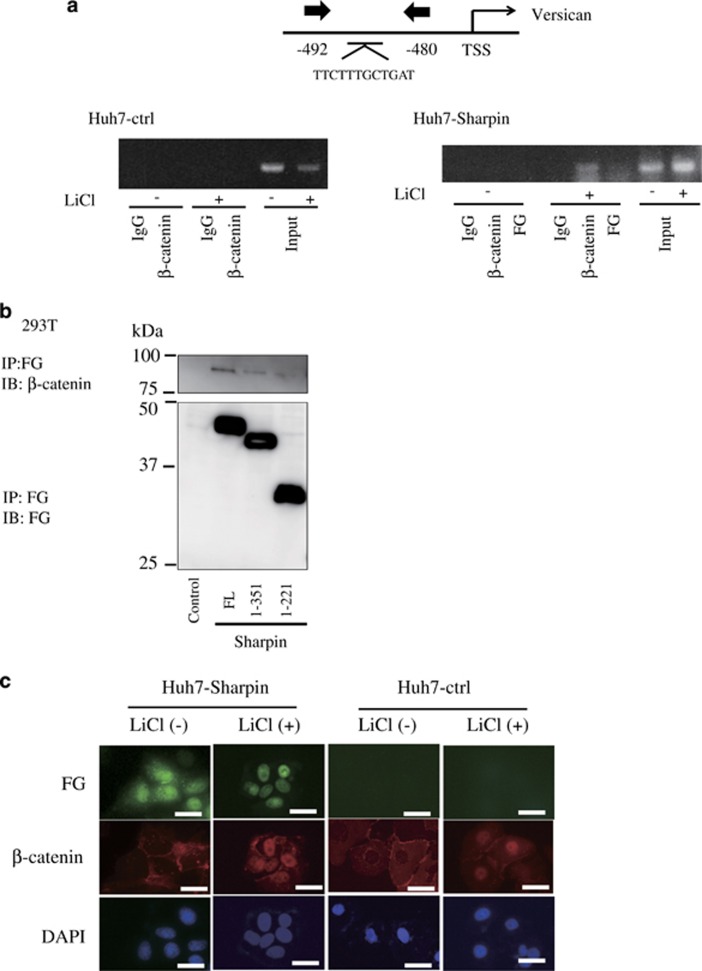
Sharpin may act as a coactivator for Versican expression. (**a**) Chromatin immunoprecipitation analysis was performed using Huh7-Sharpin or Huh7-ctrl cells 48 h after LiCl treatment. Antibodies used were mouse anti-β-catenin antibody and mouse anti-FG antibody with respective control IgG. (**b**) Sharpin interacts with β-catenin in its carboxyl terminus region. 293 T cells were transfected with FG-Sharpin or its deletion mutants Sharpin (1–351) and Sharpin (1–221), and immunoprecipitation was performed. (**c**) Subcellular localization of Sharpin and β-catenin in Huh7-Sharpin and Huh7-ctrl cells after LiCl stimulation (48 h). Localization of Sharpin (green) and β-catenin (red) are shown. The nuclei were stained with DAPI (blue). Scale bars, 10 μm.

A chromatin immunoprecipitation (ChIP) assay was performed to examine whether Versican transcription is directly regulated by Sharpin. DNA samples were prepared from Huh7-Sharpin and control cells treated with LiCl or mock (NaCl). ChIP data from control cell DNA indicated the enrichment of β-catenin, not Sharpin, in the promoter region of Versican genes, which is located 0–500 bp upstream of the transcription start site (Figure 5a).

Immunoprecipitation showed that Sharpin interacted with endogenous β-catenin (Figure 5b). Immunoprecipitation using deletion mutants of Sharpin, Sharpin (1–351) and Sharpin (1–221) showed that only full-length Sharpin bound endogenous β-catenin efficiently (Figure 5b).

Sharpin-expressing Huh7 cells stimulated with LiCl were examined by immunocytochemistry to microscopically assess the subcellular localization of Sharpin and β-catenin. As previously reported, Sharpin was localized both in the cytoplasm and in the nucleus, whereas endogenous β-catenin was mainly localized in the cytoplasm near the cell membrane (Figure 5c, Huh7-Sharpin, left).^12^ Upon stimulation with LiCl, β-catenin entered the nucleus and Sharpin was relocalized into the nucleus (Figure 5c, Huh7-Sharpin, right). Overexpression of Sharpin did not affect the localization of endogenous β-catenin with or without LiCl stimulation (Figure 5c, Huh7-ctrl). Taken together, the above observations indicate that Sharpin facilitates the recruitment of β-catenin to its promoters but not its nuclear translocation.

These results suggest that Sharpin recruits β-catenin to the Versican promoter region upon Wnt signaling activation.

### Sharpin promotes the tumorigenesis of hepatoma cells *in vivo*

We investigated whether the role of Sharpin in cell invasion significantly contributes toward tumorigenic potential of HCCs *in vivo*. Huh7-ctrl and Sharpin-expressing Huh7 cells (1 × 10^7^) were injected into nude mice via the splenic vein (*n*=6). The growth of hepatic tumors originating from Huh7-Sharpin and Huh7-ctrl cells were compared after 5 weeks.

**Figure 6:**
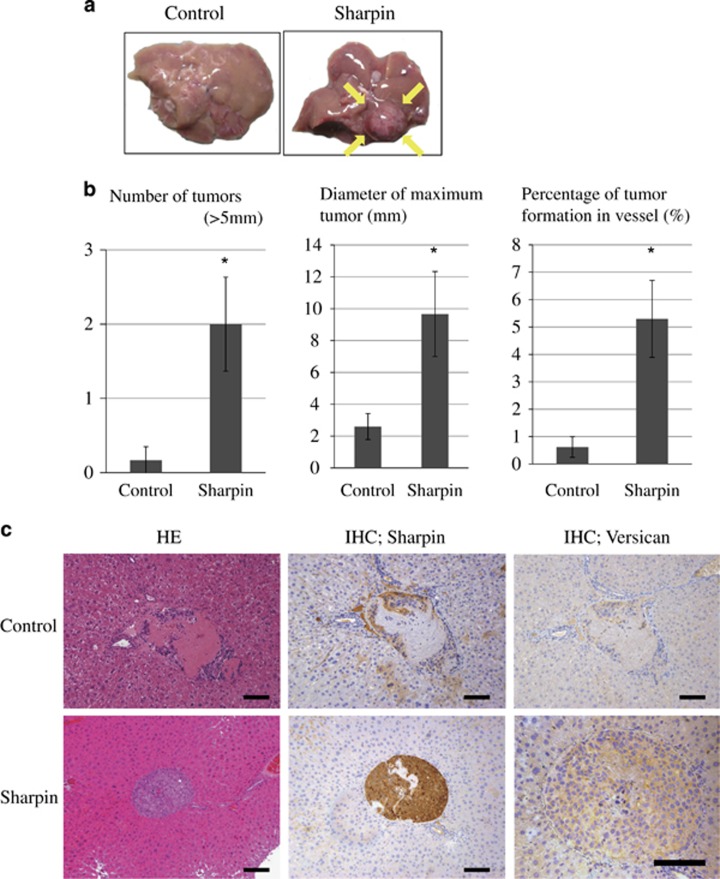
Sharpin promotes the tumorigenesis of hepatoma cells *in vivo*. (**a**) Huh7-Sharpin or Huh7-ctrl cells were injected into the splenic vein of nude mice. After 5 weeks, mice injected with Huh7-Sharpin cells developed more liver tumors than mice transplanted with Huh7-ctrl cells. Representative case is shown. Arrows indicate the liver tumor formed. (**b**) Number of tumors, diameter of maximum tumor and percentage of tumor formation in the vessel are shown (*n*=6, *P<0.05, two-tailed Student's *t*-test). (**c**) Representative images of Sharpin and Versican immunostaining of mouse tumor tissues are shown: hematoxylin and eosin-stained sections ( × 200, left), Sharpin-stained sections ( × 200, middle) and Versican-stained sections ( × 200 and × 400, right). Scale bar, 50 μm.

Tumor formation in the liver was markedly greater in mice injected with Huh7-Sharpin cells than in control mice. No visible tumor was observed in mice injected with Huh7-ctrl cells (Figure 6a). The sizes and numbers of liver tumors formed were significantly greater in mice treated with Huh7-Sharpin cells than in controls (Figure 6b). Notably, the percentage of tumor formations in the portal or hepatic vein was significantly greater in Huh7-Sharpin-treated mice than in control mice (*P*<0.05, two-tailed Student's *t*-test; Figure 6b). However, no metastatic lung nodule was observed in either group. Expression of Sharpin and Versican was confirmed by immunohistochemistry (Figure 6c).

These results suggest that Sharpin contributes to tumor formation *in vivo*.

## Discussion

An important role of Sharpin is as a component of the linear polyubiquitin chain protein complex that activates NF-κB signaling.^5, 6, 7^ However, the deletion of each molecule in the LUBAC results in a different phenotype. For instance, homozygous inactivating Sharpin mutation in mice triggers the development of severe multiorgan inflammation with a prominent chronic proliferative dermatitis phenotype.^22^ By contrast, HOIL-1 knockout mice are viable and phenotypically normal.^23^ HOIP knockout in mice causes embryonic lethality by aberrant TNFR1-mediated endothelial cell death,^24^ indicating that these genes have distinct roles in except for NF-κB signaling.

Several studies have shown that Sharpin is involved in tumor growth and invasion through NF-κB activation.^13^ We speculated that this invasive property is also NF-κB dependent. However, TNF-α stimulation did not enhance Huh7 cell invasion in our *in vitro* experimental settings. In addition, Sharpin overexpression alone did not activate NF-κB signaling. HOIL, the catalytic subunit of the LUBAC that generates the linear polyubiquitin chain, is required for full activation.^5^ It is still possible that NF-κB signaling is required for this phenomenon as the NZF and UBL domains of Sharpin are required. These domains also seem to be required for β-catenin binding for downstream activation. These results indicate that Sharpin has the ability to promote HCC invasion, at least in part through NF-κB-independent mechanisms. Although Sharpin is mainly localized in the cytoplasm, a small fraction of Sharpin is localized in the nucleus^10,12^ showing that Sharpin could function as a coactivator of a specific transcription factor. Sharpin expression alone and Wnt pathway activation induced only a modest activation of the Versican promoter. However, Sharpin expression with Wnt pathway activation synergistically enhanced Versican transcription. One possibility is that Sharpin may determine and stabilize β-catenin recruitment onto the Versican promoter region.

Versican consists of four isoforms: V0, V1, V2 and V3. Each isoform has distinct roles. V1 has been shown to have cancer-promoting functions, such as enhancing cell proliferation, inducing apoptosis resistance, inhibiting cell adhesion, and promoting cell motility.^18^ Although there are several reports of Versican in tumor invasion, the mechanisms underlying how Versican enhances invasion or metastasis remain poorly understood. One study showed that Versican acts on macrophages through TLR2/TLR6, leading to the production of inflammatory cytokines that enhance metastasis.^25^ A recent study has shown that forkhead box Q1-induced VersicanV1 expression promotes HCC metastasis.^20^ Our *in vitro* invasion assay showed that knocking down Versican in HCC without macrophages reduced HCC invasion, suggesting that it is partially independent of macrophages. Although Versican is an extracellular matrix protein, Versican is also expressed in the liver cytoplasm and functions as an invasion enhancer.^20^ Versican transcription is regulated not only by TCF, but also by p53^26^ and AP-1.^27^ However, p53 and AP-1 did not affect Versican transcription in HCC cells (data not shown), indicating that the regulation of Versican transcription is cell type-specific.

Our *in vivo* experiment provides evidence that Sharpin and Versican expression promote HCC formation, especially in either the portal vein or hepatic vein *in vivo*. Although distant lung metastasis was not observed, Sharpin increased tumor progression *in vivo*.

In conclusion, we have shown that Sharpin plays a novel role in HCC progression. A Sharpin/Versican axis could be an attractive therapeutic target for this currently untreatable cancer.

## Materials and methods

### Cell lines and transfection

The human hepatoma cell lines Huh7, Hep3B, HLE, JHH7 and SkHep1 were obtained from the Japanese Collection of Research Bioresources (Osaka, Japan). HepG2 and PLC/PRF/5 were obtained from RIKEN Cell Bank (Tsukuba, Japan) and the embryonic kidney cell line 293 T was from the American Type Culture Collection (Manassas, VA, USA)

JHH7 was maintained in William's E Medium (Sigma, St Louis, MO, USA) containing 10% heat-inactivated fetal bovine serum (FBS). Other cell cultures were maintained in Dulbecco's modified Eagle's medium (DMEM; Sigma) containing 10% heat-inactivated FBS. The cells were transfected using the Effectene Transfection Reagent (Qiagen, Hilden, Germany) according to the manufacturer's instructions.

### Antibodies and reagents

Mouse monoclonal anti-flag M2 (DYKDDDDK) antibody and its agarose-conjugated form, anti-Sharpin, and anti-β-actin were purchased from Sigma. Cell Signaling (Beverly, MA, USA) provided rabbit anti-β-catenin, Novus Biologicals (Littleton, CO, USA) provided anti-pan-Versican, and Molecular Probes (Eugene, OR, USA) provided Alexa Fluor 488 goat anti-mouse IgG secondary antibody and Alexa Fluor 555 goat anti-rabbit IgG secondary antibody. Bethyl Laboratories (Montgomery, TX, USA) provided mouse anti-β-catenin antibody. Anti-mouse and rabbit IgG, horseradish peroxidase (HRP)-linked antibodies were purchased from Amersham Biosciences (Uppsala, Sweden).

Other chemicals and reagents were from Sigma unless otherwise specified.

### Plasmids

The human Sharpin and HOIL-1L expression plasmids, pcDNA3.1 human flag-Sharpin and pcDNA3.1 human HOIL-1L-His6-HA, were kindly provided by Dr Kazuhiro Iwai (Department of Molecular and Cellular Physiology, Graduate School of Medicine, Kyoto University, Kyoto, Japan). Full-length Sharpin and its deletion fragments were amplified by PCR and subcloned into pcDNA3.1; thus, pcDNA3.1 flag-Sharpin (1–351) and flag-Sharpin (1–221) were obtained.

The wild-type and mutant Versican reporters (wt-Versican-Luc and -492mut-Versican-Luc) were kindly provided by Dr Bruce M. McManus (Department of Pathology and Laboratory Medicine, University of British Columbia, Vancouver, Canada).^21^

The lentiviral vector used for knockdowns and protein expression, pTY-shRNA-EF1a-puroR-2a-Flag, has been described previously.^28^ Full-length and deletion mutants of Sharpin were cloned into the vector, resulting in pTY-Sharpin FL, pTY-Sharpin (1–351) and pTY-Sharpin (1–221), respectively.

The plasmid pNF-κBLuc, containing the *Photinus pyralis* (firefly) luciferase reporter gene driven by a basic promoter element (TATA box) plus five repeats of the binding site for NF-κB (TGGGGACTTTCCGC), was purchased from Stratagene (La Jolla, CA, USA). The plasmid pRL-TK, featuring a *Renilla reniformis* (sea pansy) luciferase driven by the herpes simplex virus thymidine kinase promoter, was purchased from Promega (Madison, WI, USA). TOPflash/FOPflash reporter plasmid system for the detection of β-catenin-driven Wnt-transcriptional activity was described previously.^29^

### Human clinical samples

Surgically resected HCCs were used for quantitative reverse transcription-PCR (qRT-PCR) analysis. Samples were obtained from patients who underwent hepatectomy for HCC at the University of Tokyo between November 2013 and October 2014. These procedures were approved by the Ethical Committee for Clinical Research of our institution and written informed consent was obtained from each patient. The clinical diagnosis of all samples as HCC was confirmed by the Department of Pathology at the University of Tokyo Hospital.

### Quantitative reverse transcription-PCR

Total RNA was extracted from cultured cells using NucleoSpin RNAII (Takara, Tokyo, Japan). The purified RNA was reverse transcribed using the ImProm-II Reverse Transcription system (Promega) and amplified by RT-PCR. The qRT-PCR analysis was performed using a PCR mixture containing a complementary DNA sample, forward and reverse primers, and the Power SYBR Green master mix (Applied Biosystems, Foster City, CA, USA), using the ABI PRISM 7000 Quantitative PCR system (Applied Biosystems) according to the manufacturer's instructions. The amount of PCR product was normalized against GAPDH as an internal control.

The following primer pairs were used: Sharpin forward: 5′-CAACCCTCAGGAAGCTCAG-3′ and reverse: 5′-CTTGCTGCCATTCTGTCCT-3′ GAPDH forward: 5′-ATGACATCAAGAAGGTGGTG-3′ and reverse: 5′-CATACCAGGAAATGAGCTTG-3′ Versican total forward: 5′-CAAGCATCCTGTCTCACGAA-3′ and reverse: 5′-CAACGGAAGTCATGCTCAAA-3′ Versican V0 forward: 5′-GACCTCAGGCGCTTTC-3′ and reverse: 5′-CAGTGGTAACGAGATGCTTC-3′ V1 forward: 5′-GCGCCACCCTGTGAC-3′ and reverse: 5′-CAGTGGTAACGAGATGCTTC-3′ V2 forward: 5′-GACCTCAGGCGCTTTC-3′ and reverse: 5′-TAGCACTGCCCTTGGA-3′ V3 forward: 5′-TGGAGGTGGTCTACTTGG-3′ and reverse: 5′-TCACATGTCTCGGTATCTTG-3′.

### Immunoblotting

Immunoblotting of cell lysates was performed as described previously.^30^ An HRP-conjugated secondary antibody (Amersham Biosciences) was used at a 1:5000 dilution. Protein–antibody complexes were detected using ECL Plus (Amersham Biosciences).

### Immunohistochemistry

Tissue arrays containing HCC tissue and adjacent non-cancerous liver tissues were purchased from US Biomax (Rockville, MD, USA). Slides were deparaffinized and antigen retrieval was performed by incubating slides at 120 °C in 10 mM sodium citrate buffer (pH 6.0) for 20 min. To minimize nonspecific background staining, endogenous peroxidase activity was blocked by incubation in 3% hydrogen peroxide buffer for 10 min and slides were blocked in 5% normal goat serum for 10 min at room temperature. Primary antibodies were applied overnight at 4 °C and incubated with HRP-conjugated secondary antibody (Nichirei Bioscience, Tokyo, Japan) for 30 min. Antibody binding was visualized by incubation in 3,3′-diaminobenzidine in buffered substrate (Nichirei Bioscience) for 10 min. Slides were finally counterstained with hematoxylin and mounted using mounting medium. The staining intensity was classified into two categories: no or faint staining was ranked as (−), and moderate or dark staining was ranked as (+).

### Cell proliferation assay

The cell proliferation assay was performed as described in the manufacturer's protocol. Briefly, cells were seeded in 96-well plates at a density of 4 × 10^3^ cells per well. The number of viable cells in triplicate wells was determined using the Cell Counting Kit-8 (Dojindo Molecular Technologies, Rockville, MD, USA).

### Lentivirus-mediated introduction of transgene

Transgene experiments were performed as previously described using pTY-based lentiviruses expressing specific Sharpin proteins (pTY-Sharpin FL, pTY-Sharpin (1–351) and pTY-Sharpin (1–221)).^28^ We infected indicated cells with lentiviruses expressing Sharpin in the presence of 8 μg/ml polybrene. After 24 h, stably transfected cells were selected with puromycin (2 μg/ml) and the cell pool was established.

### Matrigel invasion assay

The matrigel invasion assay was performed using the Biocoat Matrigel Invasion Chamber from BD (Franklin Lakes, NJ, USA) according to the manufacturer protocol. In brief, 5 × 10^4^ cells were plated in the upper chamber in serum-free media. The bottom chamber contained DMEM media with 10% FBS. After 24 h, the bottom of the chamber insert was fixed and stained with Diff-Quick stain (Sysmex, Kobe, Japan). Cells on the stained membrane were counted under a microscope. Each membrane was divided into four quadrants and an average from all four quadrants was calculated. At least two chambers were used for each group. Each matrigel invasion assay was performed at least three times. Relative cell invasion was normalized to that of control cells.

### RNA interference-mediated transient knockdown

RNA interference (RNAi)-mediated knockdowns were performed using RNAiMax (Invitrogen) according to the manufacturer's instructions. Ambion supplied 21-bp small interfering RNA (siRNA) nucleotide sequences targeting the coding sequences of Sharpin and Versican.

Two oligonucleotides targeting the Sharpin and Versican transcripts were designed as follows: siSharpin1 (5′-GGCUGCAGGUCACACUUGATT-3′) and siSharpin2 (5′-CCACCGUGGAAGGACAGAATT-3′); siVersican1 (5′-GGAAGAUGGGCUAUACCUATT-3′) and siVersican2 (5′-GUGUUCAACCUUAAUAGUATT-3′).

### Analysis of gene expression using complementary DNA array assays

Total RNA was extracted from Huh7-Sharpin and Huh7-ctrl cells and used for GeneChip Human Genome U133 Plus 2.0 Array analysis (Affymetrix, Santa Clara, CA, USA). Probe design files and microarray data have been submitted to the National Center for Biotechnology Information Gene Expression Omnibus database under accession number GSE87149.

### Luciferase assays

Luciferase assays were performed as previously described.^31^ Briefly, ~4 × 10^4^ 293 T cells were plated into each well of a 12-well tissue culture plate (Iwaki Glass, Chiba, Japan) 24 h before transfection. The complexes, containing a total of 0.3 μg of plasmid (0.05 μg reporter plasmid, 0.01 μg pRL-TK, and 0.24 μg of other plasmids), were transiently transfected into 293 T cells. The cells were collected 48 h after transfection and assayed for luciferase activity using the PicaGene dual sea pansy system (Toyo Ink, Tokyo, Japan). Firefly luciferase activity was measured as relative light units using a luminometer (Lumat LB9507, EG and G Berthold, Bad Wildbad, Germany). pRL-TK was used to standardize transfection efficiency. Independent experiments were performed at least three times.

### Chromatin immunoprecipitation

The control and Sharpin Huh7 cells were stimulated with lithium chloride (LiCl) or mock (Sodium chloride; NaCl) and were cross-linked by incubation in 1% formaldehyde-containing medium for 10 min at 37 °C, followed by sonication to produce soluble chromatin with DNA fragments of ~500 bp. Antibodies against β-catenin (Bethyl Laboratories) and FLAG M2 beads were used to precipitate DNA fragments bound by their corresponding elements. The protein–DNA complex was collected with protein G-Sepharose beads (Invitrogen), eluted and reverse cross-linked. Following treatment with protease K, the samples were extracted with phenol–chloroform and precipitated with ethanol. The recovered DNA was resuspended in TE buffer and used for PCR amplification. The PCR primers for the target promoter are as follows: forward 5′-GCGTAAACACGCTGTAACCA-3′ and reverse 5′-GCAGGCCAGCAATAGACAAT-3′.

### Immunoprecipitation and immunoblotting

Immunoprecipitation was performed as previously described.^31^ Cells were lysed in lysis buffer ((50 mM Tris-HCl (pH 7.5), 150 mM NaCl, 0.5% (w/v) NP-40, and the protease inhibitor cocktail Complete Mini (Roche)) and centrifuged at 15,000 *g* for 20 min at 4 °C. Cellular proteins were immunoprecipitated with antibody for 1 h at 4 °C and with protein G-Sepharose (Invitrogen) for 1 h at 4 °C. After the beads were washed with wash buffer ((50 mM Tris-HCl (pH 7.5), 150 mM NaCl, 0.5% (w/v) NP-40)), the bound proteins were eluted by boiling in SDS sample buffer.

### Immunofluorescence microscopy

Huh7 cells were seeded in a slide flask (Nalge Nunc, Naperville, IL, USA) at a density of 3 × 10^4^ cells/cm^2^ and stimulated with LiCl for 48 h. The cells were then fixed with 2% paraformaldehyde for 10 min at room temperature, rinsed with PBS and permeabilized with 0.25% Triton X-100/PBS for 10 min. The cells were then rinsed with PBS and incubated with anti-FLAG M2 or β-catenin antibodies for 1 h at room temperature, followed by incubation with Alexa Fluor 488 goat anti-mouse or 555 goat anti-rabbit IgG secondary antibody (1:100 dilution) for 1 h at room temperature. Cell nuclei were stained with DAPI (Sigma). The cells were mounted with DAKO Fluorescent mounting medium (DAKO, Carpinteria, CA, USA). Fluorescence images were captured using DP2-BSW software (Olympus, Tokyo, Japan).

### *In vivo* tumorigenic experiments

BALB/c nude mice (8 weeks old, male) were purchased from CLEA Corp. (Tokyo, Japan). All procedures involving experimental animals were performed in accordance with protocols approved by the Institutional Committee for Animal Research of the University of Tokyo and complied with the USPHS Policy on Humane Care and Use of Laboratory Animals. Huh7 cells (1 × 10^7^) were injected into the splenic vein of six nude mice. After 5 weeks, the mice were anesthetized with pentobarbital sodium (120 mg/kg) and tumor formation in the liver was analyzed. The experiments were performed in a non-randomized and non-blinded manner.

### Statistics

All numerical values are expressed as the mean±s.d. of at least three independent experiments. Spearman's analysis of correlation analyses, Wilcoxon signed-rank tests, two-tailed Student's *t*-tests, and Fisher's exact probability tests were used for statistical analysis. Values of *P*<0.05 were deemed to indicate statistical significance.

## Figures and Tables

**Table 2:** Increased expression of genes in stably Sharpin-expressing Huh7 cells compared with wild-type cells

*Representative public ID*	*Gene symbol*	*Gene title*	*Log2 ratio*
AL137717	ANKRD36BP2	Ankyrin repeat domain 36B pseudogene 2	6.50
AI770005	PKHD1	Polycystic kidney and hepatic disease 1 (autosomal recessive)	5.73
BC041438	—	—	5.58
D32039	VCAN	Versican	5.58
BC035679	—	—	5.44
BC040319	LOC339622	Uncharacterized LOC339622	5.39
AI378647	F2RL2	Coagulation factor II (thrombin) receptor-like 2	5.14
BC022036	LOC401497	Uncharacterized LOC401497	5.09
NM_004437	EPB41	Erythrocyte membrane protein band 4.1 (elliptocytosis 1, RH-linked)	5.09
AI138237	C20orf62	Chromosome 20 open reading frame 62	5.00

**Table 1:** Spearman's analysis of correlation between Sharpin expression and clinico-pathological parameters

*Variable*	*ρ*	P-*value*
Tumor size	0.4015	0.0342
Histological grading	0.5323	0.0043
