# Supplementary material for: Sharpin promotes hepatocellular carcinoma progression via transactivation of Versican expression
Source: Oncogenesis. 2016 Dec 12;5(12):e277–. doi: 10.1038/oncsis.2016.76 (PMC5177774; doi:10.1038/oncsis.2016.76)
Supplement: Supplementary Material [file oncsis201676x1.docx]

**Supplementary information to:**

**Sharpin promotes hepatocellular carcinoma progression via transactivation of versican expression**

Yasuo Tanaka, Keisuke Tateishi, Takuma Nakatsuka, Yotaro Kudo,

Ryota Takahashi, Koji Miyabayashi, Keisuke Yamamoto, Yoshinari Asaoka,

Hideaki Ijichi, Ryosuke Tateishi, Junji Shibahara, Masashi Fukayama,

Takeaki Ishizawa, Kiyoshi Hasegawa, Norihiro Kokudo, and Kazuhiko Koike

**Supplementary Figure S1. Proliferation assay revealed no significant difference in cell growth between control and Sharpin-expressing cells**

Huh7-Sharpin and control cells were seeded in 96-well plates at a density of 4

× 10^3^ cells per well. The number of viable cells in triplicate wells was determined using

the Cell Counting Kit-8 at days 1, 2, and 3. Data represent the mean ± SD (N.S.: not significant, two-tailed Student’s t-test).

**Supplementary Figure S2. The effect of Sharpin knockdown in Huh7 cells for invasive property**

Endogenous Sharpin was knocked down in Huh7 cells and an invasion assay was performed 48 h later. Representative images of stained invaded cells (left). The relative cell invasion ratio after normalization to control cell invasion levels (middle). Expression level of Sharpin mRNA in Huh7 cells are shown (right). Data represent the mean ± SD (N.S.: not significant, * p < 0.05, two-tailed Student’s t-test).

**Supplementary Figure S3. TNF α stimulation did not enhance HCC invasion**

Huh7-Sharpin and control cells were established using a lentiviral vector and the degree of cell invasion was examined using Matrigel cell invasion chambers with TNF-α stimulation (50 ng/ml). The relative cell invasion ratio after normalization to control cell invasion levels is shown. Data represent the mean ± SD (N.S.: not significant, two-tailed Student’s t-test).

**Supplementary Figure S4. Knockdown of Versican inhibited invasion of HCC cells.**

Versican was knocked down in HepG2 cells and an invasion assay was performed. Representative images of stained invaded cells (left). The relative cell invasion ratio after normalization to control cell invasion levels (middle). Versican expression was determined by qRT-PCR (right). Data represent the mean ± SD (* p < 0.05, two-tailed Student’s t-test).

**Supplementary Figure S5. Overexpression of Sharpin alone did not induce NF-κB activation**

293T cells were cotransfected with pNF-κBLuc and Myc-HOIP, FG-Sharpin

and its deletion mutants (FG-Sharpin (1–351) and (1–221)) expression vector. Cell

lysates were collected 48 h later and luciferase assays were performed. Values were

internally normalized to Renilla (mean ± SD) (* p < 0.05, two-tailed Student’s t-test).
