# Supplementary figures and images for: Sharpin promotes hepatocellular carcinoma progression via transactivation of Versican expression
Source: Oncogenesis. 2016 Dec 12;5(12):e277–. doi: 10.1038/oncsis.2016.76 (PMC5177774; doi:10.1038/oncsis.2016.76)

Supplementary Figure S1.

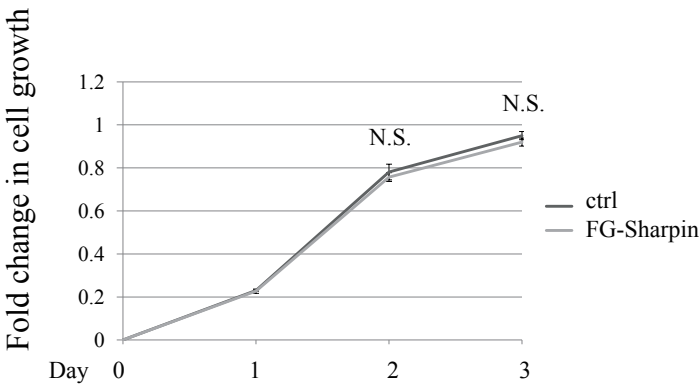

Supplement: Supplementary Figure 1 [file oncsis201676x2.pdf]

Supplementary Figure S2.

Huh7

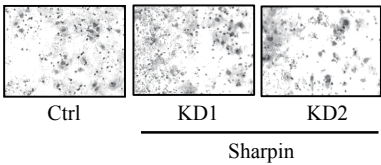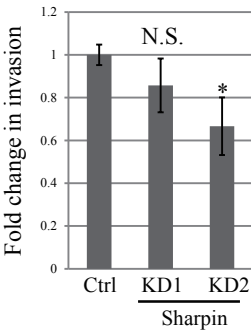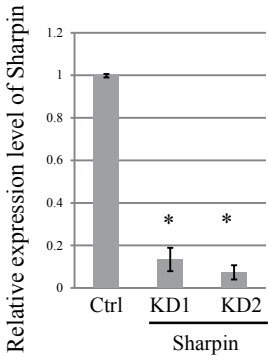

Supplement: Supplementary Figure 2 [file oncsis201676x3.pdf]

Supplementary Figure S3.

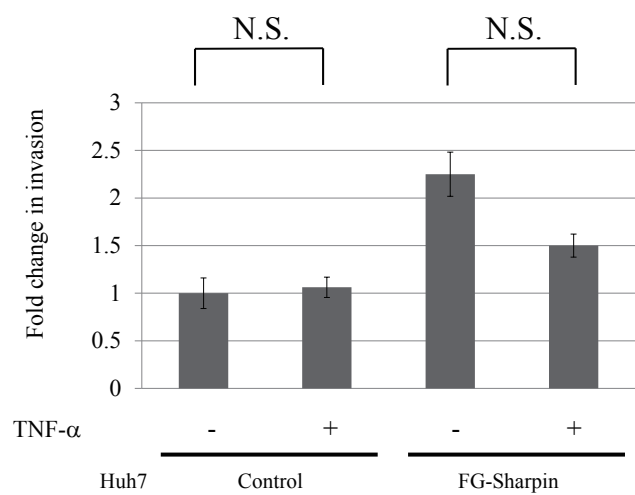

Supplement: Supplementary Figure 3 [file oncsis201676x4.pdf]

Supplementary Figure S4.

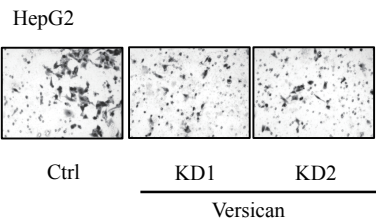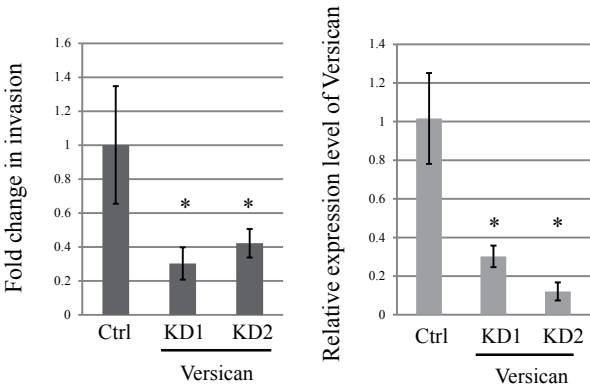

Supplement: Supplementary Figure 4 [file oncsis201676x5.pdf]

Supplementary Figure S5.

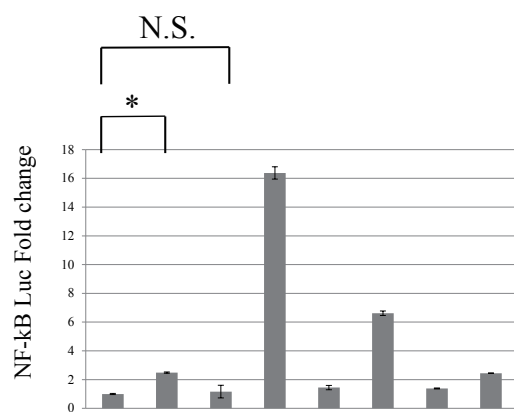

|                    |   |   |   |   |   |   |   |   |
|--------------------|---|---|---|---|---|---|---|---|
| Myc-HOIP           | - | + | - | + | - | + | - | + |
| FG-Sharpin WT      | - | - | + | + | - | - | - | - |
| FG-Sharpin (1-351) | - | - | - | - | + | + | - | - |
| FG-Sharpin (1-221) | - | - | - | - | - | - | + | + |

Supplement: Supplementary Figure 5 [file oncsis201676x6.pdf]
